# The cytotoxicity of gallium maltolate in glioblastoma cells is enhanced by metformin through combined action on mitochondrial complex 1

**DOI:** 10.18632/oncotarget.27567

**Published:** 2020-04-28

**Authors:** Hisham S. Alhajala, John L. Markley, Jin Hae Kim, Mona M. Al-Gizawiy, Kathleen M. Schmainda, John S. Kuo, Christopher R. Chitambar

**Affiliations:** ^1^ Division of Hematology and Oncology, Department of Medicine, Medical College of Wisconsin, Milwaukee, Wisconsin, USA; ^2^ Department of Biochemistry, University of Wisconsin-Madison, Madison, Wisconsin, USA; ^3^ Department of Biophysics, Medical College of Wisconsin, Milwaukee, Wisconsin, USA; ^4^ Department of Neurosurgery and Mulva Clinic for the Neurosciences, Dell Medical School, Austin, Texas, USA

**Keywords:** glioblastoma, iron, gallium, metformin, mitochondrial complex I

## Abstract

New drugs are needed for glioblastoma, an aggressive brain tumor with a dismal prognosis. We recently reported that gallium maltolate (GaM) retards the growth of glioblastoma in a rat orthotopic brain tumor model by inhibiting mitochondrial function and iron-dependent ribonucleotide reductase (RR). However, GaM’s mechanism of action at the mitochondrial level is not known. Given the interaction between gallium and iron metabolism, we hypothesized that gallium might target iron-sulfur (Fe-S) cluster-containing mitochondrial proteins. Using Extracellular Flux Analyzer technology, we confirmed that after a 24-h incubation, GaM 50 μmol/L inhibited glioblastoma cell growth by <10% but inhibited cellular oxygen consumption rate by 44% and abrogated mitochondrial reserve capacity. GaM blocked mitochondrial complex I activity and produced a 2.9-fold increase in cellular ROS. NMR spectroscopy revealed that gallium binds to IscU, the bacterial scaffold protein for Fe-S cluster assembly and stabilizes its folded state. Gallium inhibited the rate of *in vitro* cluster assembly catalyzed by bacterial cysteine desulfurase in a reaction mixture containing IscU, Fe (II), DTT, and L-cysteine. Metformin, a complex I inhibitor, enhanced GaM’s inhibition of complex I, further increased cellular ROS levels, and synergistically enhanced GaM’s cytotoxicity in glioblastoma cells in 2-D and 3-D cultures. Metformin did not affect GaM action on cellular iron uptake or transferrin receptor1 expression nor did it enhance the cytotoxicity of the RR inhibitor Didox. Our results show that GaM inhibits complex I by disrupting iron-sulfur cluster assembly and that its cytotoxicity can be synergistically enhanced by metformin through combined action on complex I.

## INTRODUCTION

Glioblastoma accounts for approximately 15% of all central nervous system (CNS) tumors and approximately 47% of all malignant CNS tumors [1]. In 2018, an estimated 12,760 new cases of glioblastoma were diagnosed in the US [1]. Current treatment for this malignancy has not changed in several years and focuses primarily on maximal surgical resection followed by radiation and temozolomide chemotherapy [2]. More recently, delivery of alternating tumor-treating electric field was shown to prolong patient survival by a few months and FDA-approved as an additional glioblastoma therapy [3]. Nonetheless, outcomes in this disease are dismal; the median survival is reported to be approximately 14 months from diagnosis [4], while the one-year and five-year survival is approximately 40% and 5.5%, respectively [1]. There is thus a great need to develop additional efficacious therapies.

The present study was prompted by our recent discovery that gallium maltolate (GaM) inhibits the growth of glioblastoma cells *in vitro* and *in vivo* in an orthotopic brain tumor rodent model with established glioblastoma [5]. We showed that GaM’s mechanism of antineoplastic action included disruption of tumor iron homeostasis, an inhibition of iron-dependent ribonucleotide reductase (RR), and a decrease mitochondrial function at early time-points that preceded the onset of cell death [5]. In the present study, we sought to gain a deeper understanding of how GaM perturbs mitochondrial function and to explore whether other inhibitors of mitochondrial function could enhance its cytotoxicity. Since gallium shares certain chemical properties with iron which enable it to interact with iron-binding proteins and interfere with iron utilization by malignant cells [6], we hypothesized that GaM could disrupt the function of proteins of citric acid cycle and the mitochondrial electronic transport chain that contain iron-sulfur (Fe-S) clusters as essential cofactors.

There is a great interest in repurposing metformin [a drug used for Type 2 diabetes mellitus (T2DM)] for the treatment of cancer [7, 8]. Preclinical studies have shown metformin to have antineoplastic activity *in vitro* and in certain animal tumor models [9, 10]. With specific regard to glioblastoma, recent studies demonstrated that metformin delayed the growth of human glioblastoma cell xenograft in athymic mice and, when combined with temozolamide or with radiation therapy, synergistically inhibited the growth of glioblastoma cell lines [11]. At this writing, there are 342 cancer clinical trials listed in ClinicalTrials. gov (https://clinicaltrials.gov) in which metformin is being evaluated as a single agent, as an adjunct to conventional chemotherapy, or for cancer prevention.

One of the challenges to the success of metformin as an anticancer drug in the clinic is that the concentrations of metformin used to inhibit the growth of malignant cells *in vitro* is far greater than the plasma levels attained in diabetic patients treated with this drug [12]. However, there are other potential strategies to boost metformin’s antineoplastic action that could be explored. Since metformin is an inhibitor of mitochondrial complex 1 [13, 14] and is known to accumulate 100 to 500-fold in the mitochondria [12], combining it with other agents that target the mitochondria may enable it to exert an antitumor activity at lower doses.

Based on our knowledge of GaM’s action on the mitochondria and the fact that metformin is a known inhibitor of complex 1, we hypothesized that both drugs in combination at lower concentrations might enhance each other’s antineoplastic activity in glioblastoma. Our studies show for the first time that GaM inhibits mitochondrial function by interfering with the Fe-S assembly mechanism necessary for the activity of complex I and that both GaM and metformin in combination synergistically inhibit the proliferation of glioblastoma cell lines and glioblastoma stem cells *in vitro.* Phase 1 clinical trials of oral GaM have been conducted healthy individuals and cancer patients [15, 16], while metformin is used clinically to treat patients with T2DM. Hence, our results have potential clinical implications for glioblastoma and warrant further investigation.

## RESULTS

### GaM inhibits glioblastoma cell proliferation and inhibits mitochondrial complex I leading to an increase in intracellular ROS

**Figure 1:**
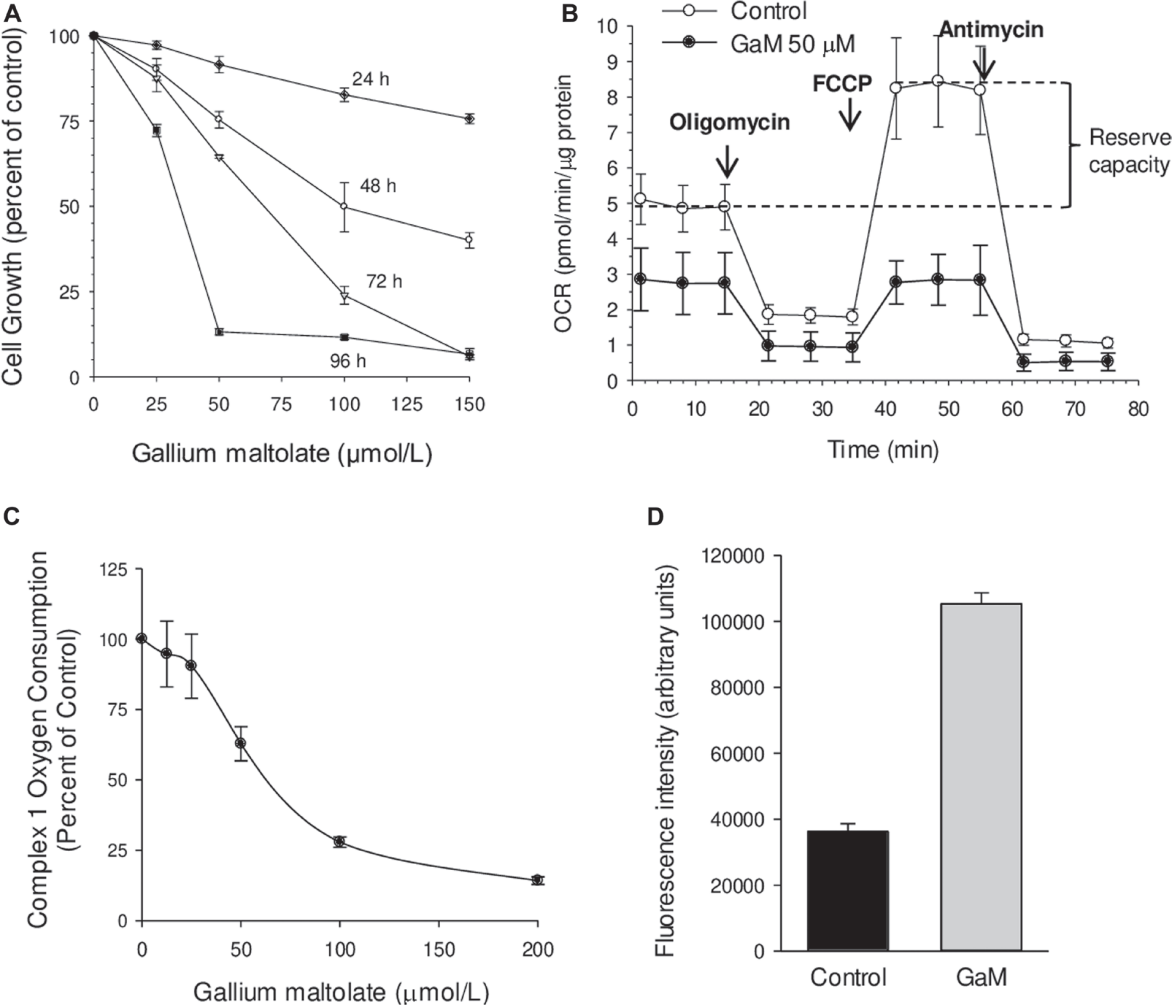
Effects of GaM on glioblastoma cell proliferation, mitochondrial function, and ROS production (**A**). Time and concentration-dependent inhibition of D54 glioblastoma cells by GaM. Cellular proliferation was measured by MTT assay. Values represent means ± S. E (*n* = 3). (**B**) Effect of GaM on mitochondrial bioenergetics in D54 cells after a 24-h incubation. Cellular oxygen consumption rate (OCR) was measured by a Seahorse XF Extracellular Flux Analyzer, as described under Methods. (**C**) GaM inhibits complex I activity. Complex I-mediated respiratory activity (OCR) was measured in D54 cells after a 24-h incubation with increasing concentrations of GaM. (**D**) GaM increases ROS production. D54 cells were analyzed for DCF-AM fluorescence after 4 h of incubation without or with GaM 50 μmol/L, as described under Methods.

Our initial experiments focused on confirming that GaM inhibited glioblastoma cell proliferation and mitochondrial function and then further elucidating the mechanism by which GaM blocks mitochondrial function. Figure 1A shows that GaM inhibited the proliferation of D54 glioblastoma cells in a dose and time-dependent manner. Although cells exposed to 50 μmol/L GaM displayed less than a 10% decrease in their growth at 24 h compared to control cells, their basal cellular oxygen consumption rate (OCR, a measure of mitochondrial function) at this time-point was decreased by approximately 44% (Figure 1B). In addition, these GaM-treated cells displayed complete loss of reserve capacity. As shown in Figure 1B, the addition of the uncoupling agent FCCP to control cells produced an increase in OCR above baseline; this measure represents the reserve capacity or spare respiratory capacity of these cells. In contrast, GaM-treated cells, FCCP failed to produce an increase in OCR above baseline (Figure 1B). The loss of reserve capacity following GaM exposure is thus an initial event that occurs before a diminution in cell proliferation or cell death can be detected.

To better understand the basis for GaM-induced inhibition of mitochondrial function, we examined its action on complex I of the electron transport chain (ETC). Complex I activity maintains NAD^+^ levels and the NAD+/NADH ratio in the mitochondrial matrix; it is the point of entry of electrons into the ETC which, in turn, drives aerobic cellular respiration. Recent studies indicate that complex I may also contribute to malignant cell growth and the induction of tumor metastases; this makes it a potential target for anticancer therapy [reviewed in reference [17]]. The effect of GaM on oxygen consumption by complex I was measured by XF Analyzer using specific substrates and inhibitors of this complex. As shown in Figure 1C, GaM, in a dose-dependent manner, inhibited the activity of complex I and produced a 2.9-fold increase in cellular DCF fluorescence relative to control cells (Figure 1D). The latter is indicative of a GaM-induced increase in cellular ROS and is consistent with the known consequences of inhibiting complex I [18].

### Fe-S cluster assembly

**Figure 2:**
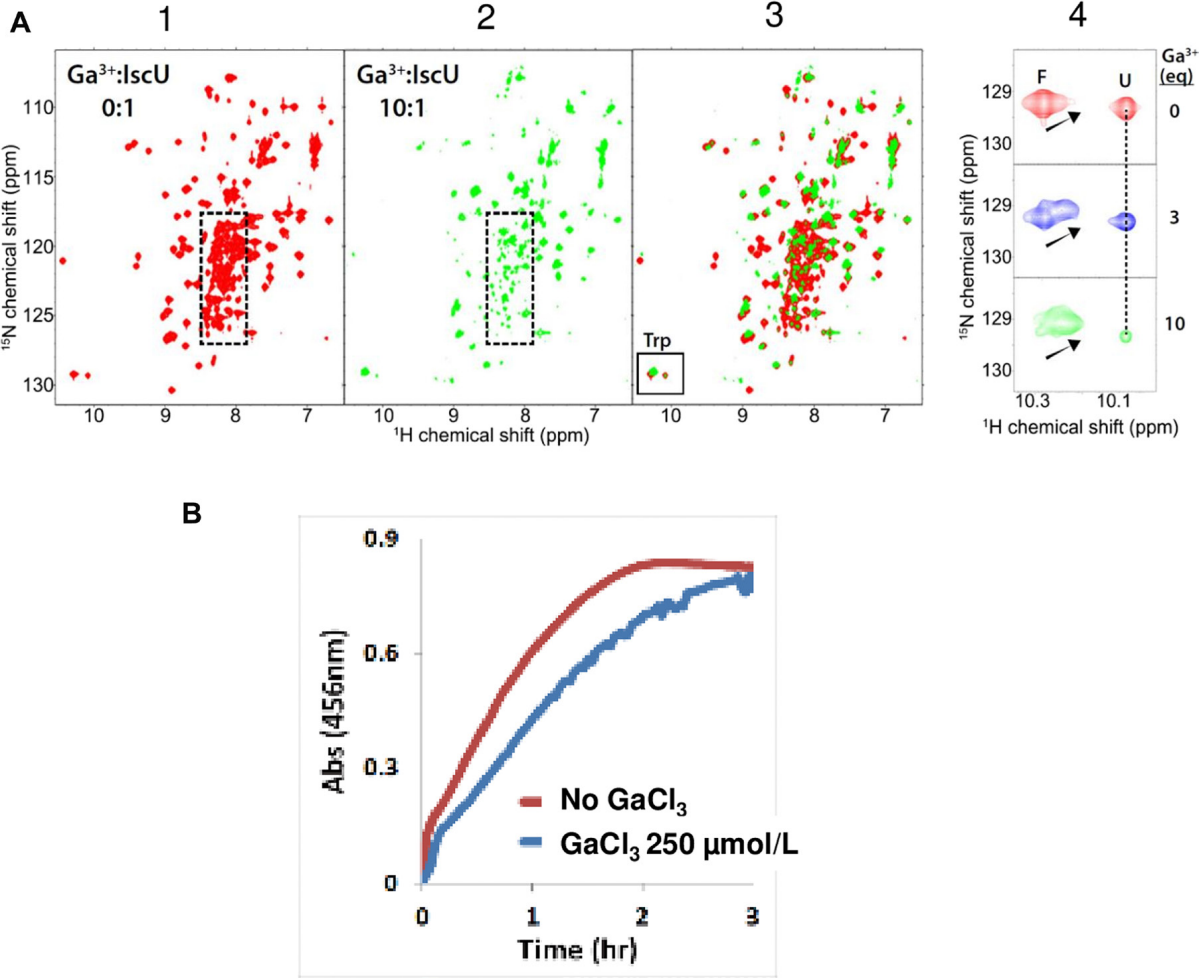
GaM interferes with the Fe-S cluster assembly machinery (**A**). Gallium binds the Fe-S cluster scaffold protein IscU. Panel 1. The ^1^H-^15^N HSQC spectrum of [U-^15^N]-IscU in 50 mM Tris buffer at pH 7.5 containing 5 mM DTT. The spectrum indicates that the protein exists as a mixture of folded and unfolded states. The highly overlapped peaks within the dotted rectangle are mainly from the unfolded state. Panel 2. The ^1^H-^15^N HSQC spectrum of [U-^15^N]-IscU following the addition of a 10-fold excess of GaCl_3_ (green) indicates that binding of Ga (III) has shifted the equilibrium toward the folded state. Signals from the unfolded protein within the dotted rectangle are weaker. Panel 3. Overlay of the ^1^H-^15^N HSQC spectra of [U-^15^N]-IscU in the absence of Ga (III) (red) and in the presence of 10-fold excess GaCl_3_ (green). The box contains signals from the side chain NH of the single tryptophan residue which reports on the relative populations of the folded and unfolded states. Panel 4. Expanded view of the tryptophan signal of IscU as a function of added GaCl_3_. The signal labeled F corresponds to protein in the folded state, and the signal labeled U corresponds to protein in the unfolded state. The results show that the addition of GaCl_3_ leads to a shift in the equilibrium between the two states toward the folded state. NMR spectra were acquired with a Varian NMR spectrometer operating at 600 MHz (^1^H). (**B**) Gallium has an inhibitory effect on the *in vitro* Fe-S cluster assembly reaction. Figure shows the time-course of iron-sulfur cluster assembly as monitored by absorbance at 456 nm. The presence of 250 μM GaCl_3_ in the reaction mixture led to inhibition of the cluster assembly rate. The cluster assembly reaction was carried out in an anaerobic chamber (Coy Laboratory) filled with 90% N_2_ and 10% H_2_ at room temperature. The O_2_ level was maintained at less than 5 ppm. The reaction mixture consisted of 50 μM IscU, 250 μM ferrous ammonium sulfate, 5 mM DTT, and 1 μM *E. coli* cysteine desulfurase (IscS) in 0.1 M Tris∙HCl buffer at pH 7.5. The reaction was initiated by adding L-cysteine to achieve a concentration of 250 μM. The reaction was carried out in a 1-cm path-length cuvette sealed with a rubber septum. A UV-1700 UV-visible spectrophotometer (Shimadzu) equipped with a temperature-controlled cell positioner was used for the absorbance measurements, and the raw data were processed with UV Probe 2.21 software (Shimadzu).

Fe-S cluster biogenesis is a complex process; it requires the coordination of proteins that donate sulfur, iron and electrons, a scaffold protein that serves as a platform for cluster assembly, and other proteins that transfer the labile scaffold-bound cluster to the proper acceptor site [19]. In this process, mitochondrial Fe-S clusters are transiently assembled on the scaffold protein IscU and then transferred to a recipient apoprotein, such as the respiratory complexes or aconitase. Delivery of Fe-S clusters to these proteins requires the scaffold protein to cycle between folded and unfolded states in response to the binding of metals and other chaperone proteins. Our protein NMR studies conducted in a bacterial system (Figure 2A) revealed that Ga (III) preferentially stabilizes the structured form of the protein. Previous studies suggested that stabilizing the structured state of IscU is correlated with a reduced rate of cluster assembly [20]. To test this hypothesis, we examined the effect of added Ga (III) on an *in vitro* cluster assembly reaction (Figure 2B). The results confirmed that Ga (III) inhibits the rate of cluster assembly.

### Inhibition of mitochondrial complex I by GaM can be increased by metformin

**Figure 3:**
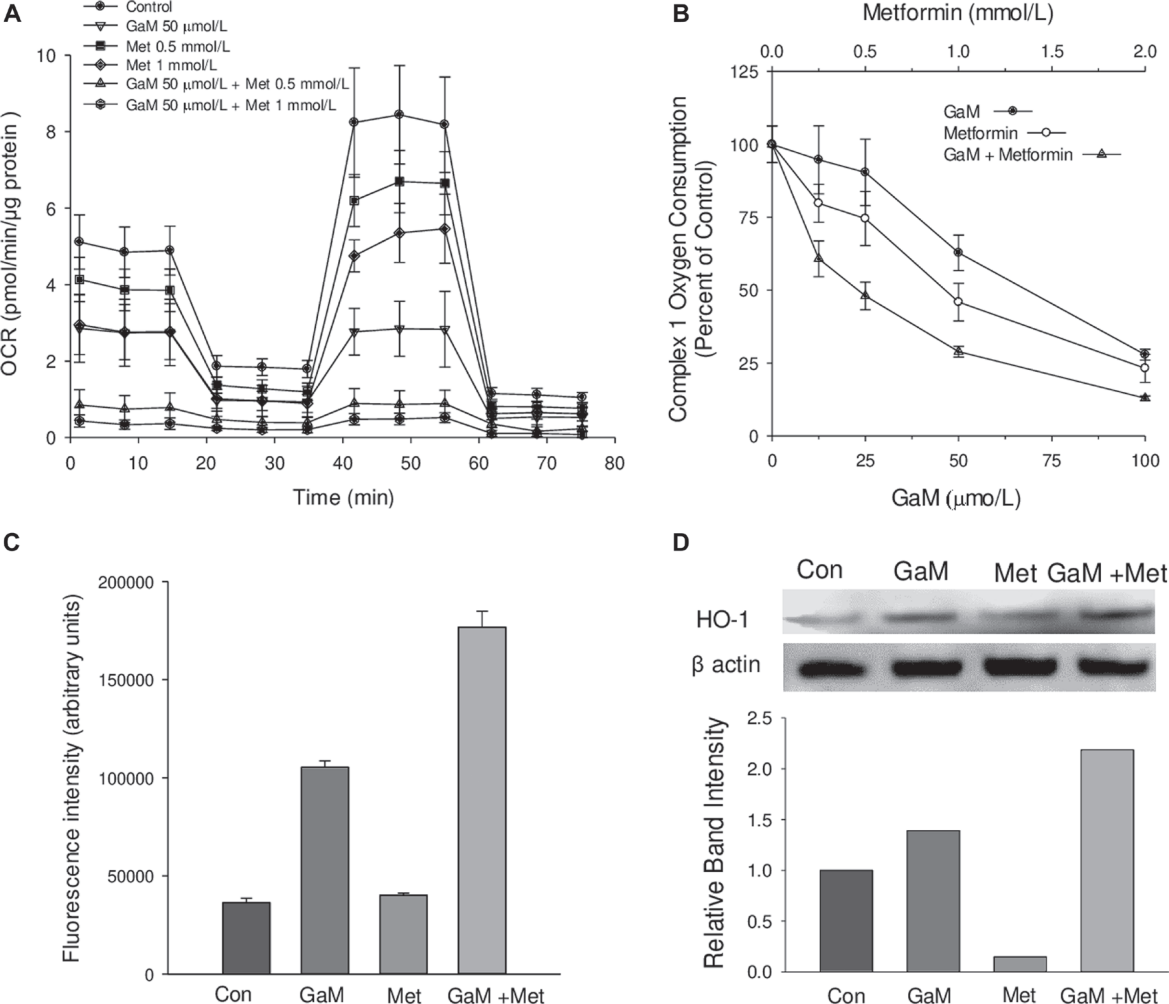
GaM inhibition of mitochondrial function is augmented by metformin (**A**). Cellular oxygen consumption rates (OCR) were measured by the Seahorse XF Analyzer, as shown in Figure 1. OCR in D54 cells was measured after a 24 h incubation with GaM 50 μmol/L, metformin 0.5 or 1.0 mmol/L, or the combination of both agents as shown in the Figure. (**B**) Inhibition of complex 1 activity by GaM and metformin alone and in combination. Cells were analyzed after a 24 h incubation with GaM and metformin. (**C**) Metformin increases GaM-induced ROS production in D54 cells. Cells were assayed for DCF fluorescence as described under Methods. Cells were analyzed for DCF-AM fluorescence as described in Figure 1. (**D**) GaM induction of heme oxygenase-1 (HO-1) expression is enhanced by metformin. HO-1 expression in cells was measured by Western Blotting after 24 h incubation of D54 cells with GaM and metformin.

Having found that GaM inhibited complex I, we examined whether its cytotoxicity could be enhanced by combining it with other agents. Metformin, an oral antihyperglycemic drug used clinically for the treatment of type 2 diabetes mellitus [8], has recently been shown to have antineoplastic activity, and there is considerable interest in repurposing it for cancer treatment [7]. Since metformin’s mechanism of antineoplastic action includes inhibition of complex I [13], we sought to determine whether this drug might enhance gallium-induced inhibition of suppression of complex I activity. Indeed, this was found to be the case. As shown in Figure 3A, combinations of GaM and metformin at molar ratios of 1:10 and 1:20 produced greater decreases in cellular OCRs than either agent alone. Specific measurements of complex I oxygen consumption showed that whereas GaM and metformin individually produced a dose-dependent decrease in complex I activity, the inhibition of complex I was significantly greater when both agents were combined (Figure 3B). Consistent with combined inhibition of complex I, cells displayed a greater level of ROS production compared with either agent alone (Figure 3C).

In prior studies, we showed that gallium nitrate increased ROS levels in human lymphoma CCRF-CEM cells leading to an upregulation of heme oxygenase-1 (HO-1) [21]. Similarly, GaM produced an increase in HO-1 levels in D54 cells; this was further increased by metformin (Figure 3D). These results are consistent with leakage of ROS from the mitochondria known to occur when complex I activity is blocked [22] and they provide additional evidence that GaM and metformin act in concert to inhibit complex I.

### GaM and metformin act synergistically to inhibit the proliferation of glioblastoma cells

**Figure 4:**
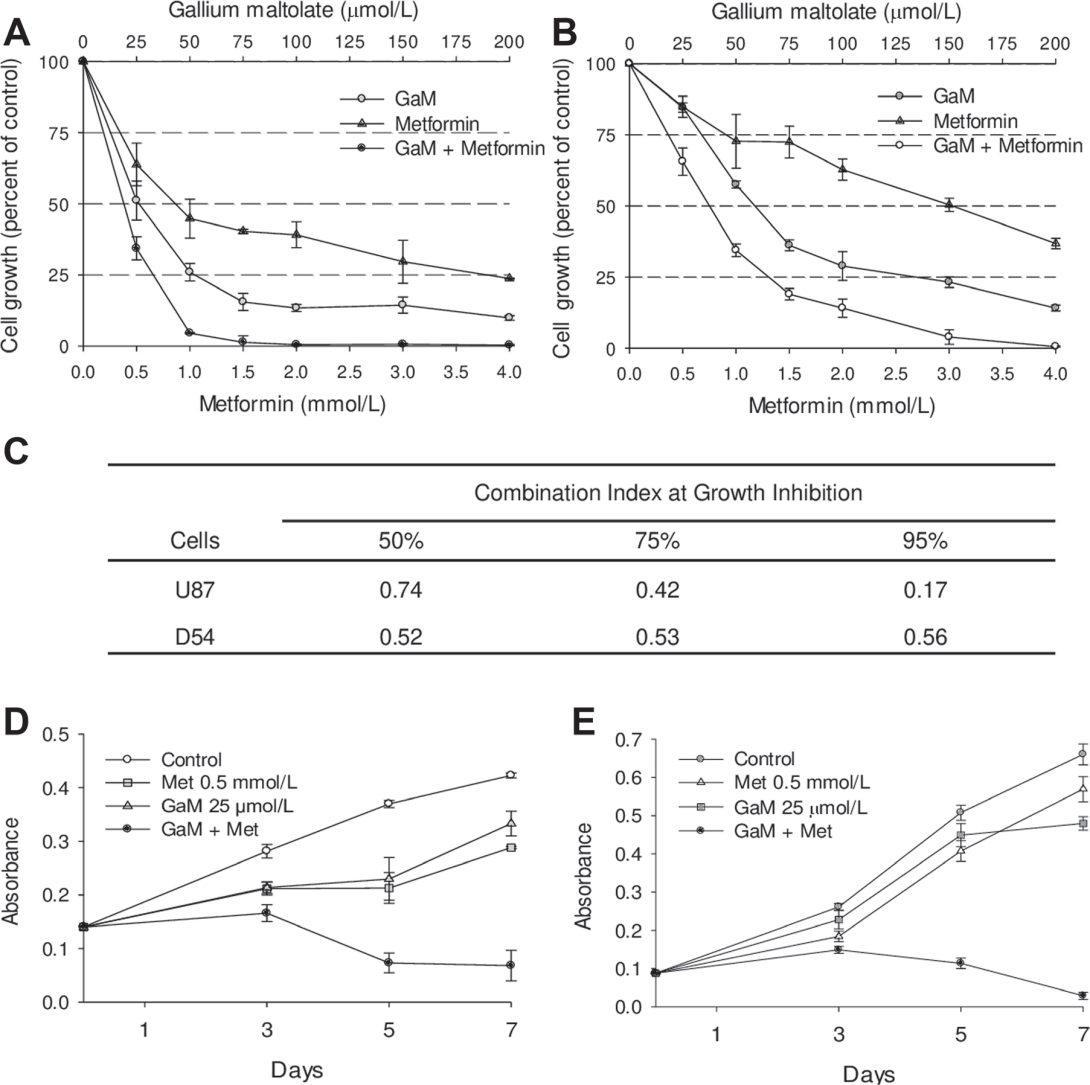
GaM and metformin synergistically inhibit the proliferation of glioblastoma cell lines (**A** and **B**). U87 MG (panel A) and D54 (panel B) cells were incubated with increasing concentrations of GaM and metformin alone and in combination at a fixed molar ratio of 1:20 (GaM: metformin). Cell proliferation was measured by MTT assay after 96 h incubation. (**C**) Combination Indices (CI). Analysis of the dose-response curves from A and B. A CI of <1 represents drug synergy between GaM and metformin. (**D** and **E**) Cell growth over time. U87 MG (panel D) and D54 (panel E) cells were plated in the absence or presence of GaM or metformin alone and in combination. Cell proliferation relative to initial plating density was measured by MTT assay after 3, 5, and 7 days of incubation.

To determine whether the inhibitory effects of GaM and metformin on complex I interpreted into an effect on cell proliferation, we examined the impact of these agents on the growth of glioblastoma cells. Dose-response studies showed that whereas both agents individually inhibited the growth of U87 MG and D54 cells, the inhibition of cell growth was significantly greater when they were combined (Figure 4A and 4B, respectively). Analysis of drug-drug interaction using the strict pharmacologic criteria of Chou and Talalay [23] confirmed that cell growth-inhibition by combination GaM and metformin was synergistic as evidenced by combination index of <1 (Figure 4C).

The effect of GaM and metformin on cell growth over an extended time was examined. U87 MG and D54 cells were incubated with the lowest concentrations of GaM (25 μmol/L) and metformin (0.5 mmol/L) used in Figures 4A and 4B, and their proliferation was determined after 3, 5, and 7 days of incubation. As shown in Figure 4D, control U87 MG cells displayed unrestricted cell growth with a progressive increase in number at each time-point while the proliferation rate of GaM- and metformin-treated cells was slower than control cells with a plateau in the growth curve between days 3 and 5 followed by an increase in cell number (albeit lower than control cells) by day 7. In contrast, cell growth in the presence of combination of GaM and metformin decreased below initial plating density by day 5 and did not recover thereafter. A similar pattern of cell proliferation was seen in control, GaM-, and metformin-treated D54 cells (Figure 4E). Collectively, these studies suggest that at the lower concentrations used, GaM and metformin slowed the rate of cell growth; in combination however, they clearly induced cell death.

**Figure 5:**
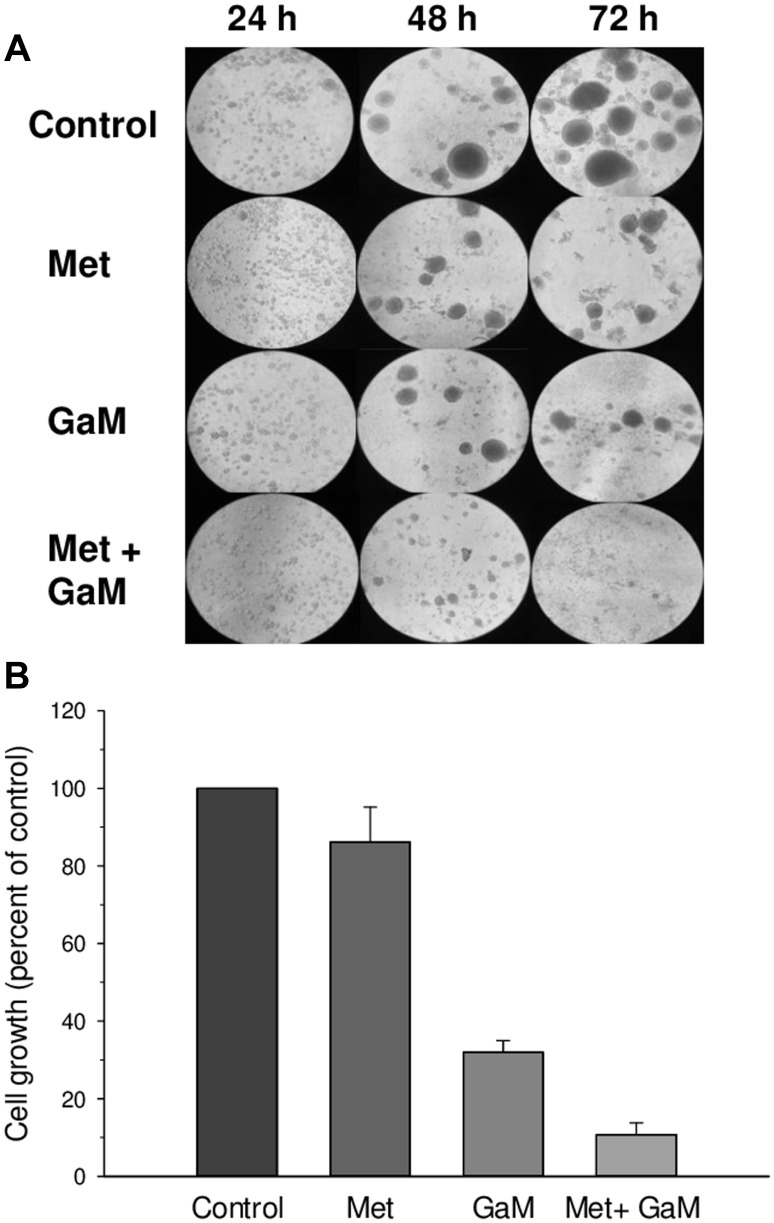
GaM and metformin inhibit the growth of glioblastoma stem cells (GSCs) (**A**). Photomicrographs of GSC-44 cell growth and neurosphere formation under different conditions. Control GSC-44 cells were plated as a single cell suspensions in culture and developed neurospheres over 48 – 72 h. Neurosphere formation was reduced in the presence of metformin (1 mmol/L) or GaM (50 μmol/L); neurosphere formation was completely blocked by the combination of metformin and GaM. (**B**) GSC-44 growth measured by MTT assay at 72 h.

In addition to glioblastoma cell lines, we examined the effect of GaM and metformin on the growth of human patient-derived GSCs using the GSC-44 line which grows as neurospheres in culture and generates highly invasive orthotopic tumor xenografts as previously described [24]. As shown in Figure 5A, analysis by light microscopy showed that single cell suspensions of these cells proliferated and formed neurospheres over the course of 72 h, while incubation with GaM or metformin alone resulted in a decrease in the size and number of neurospheres. GaM and metformin in combination completely inhibited cell growth and neurosphere formation. These findings were confirmed by proliferation assay, which showed that cell proliferation was inhibited by combination GaM and metformin (Figure 5B).

### Metformin does not increase GaM cytotoxicity by acting on iron transport

**Figure 6:**
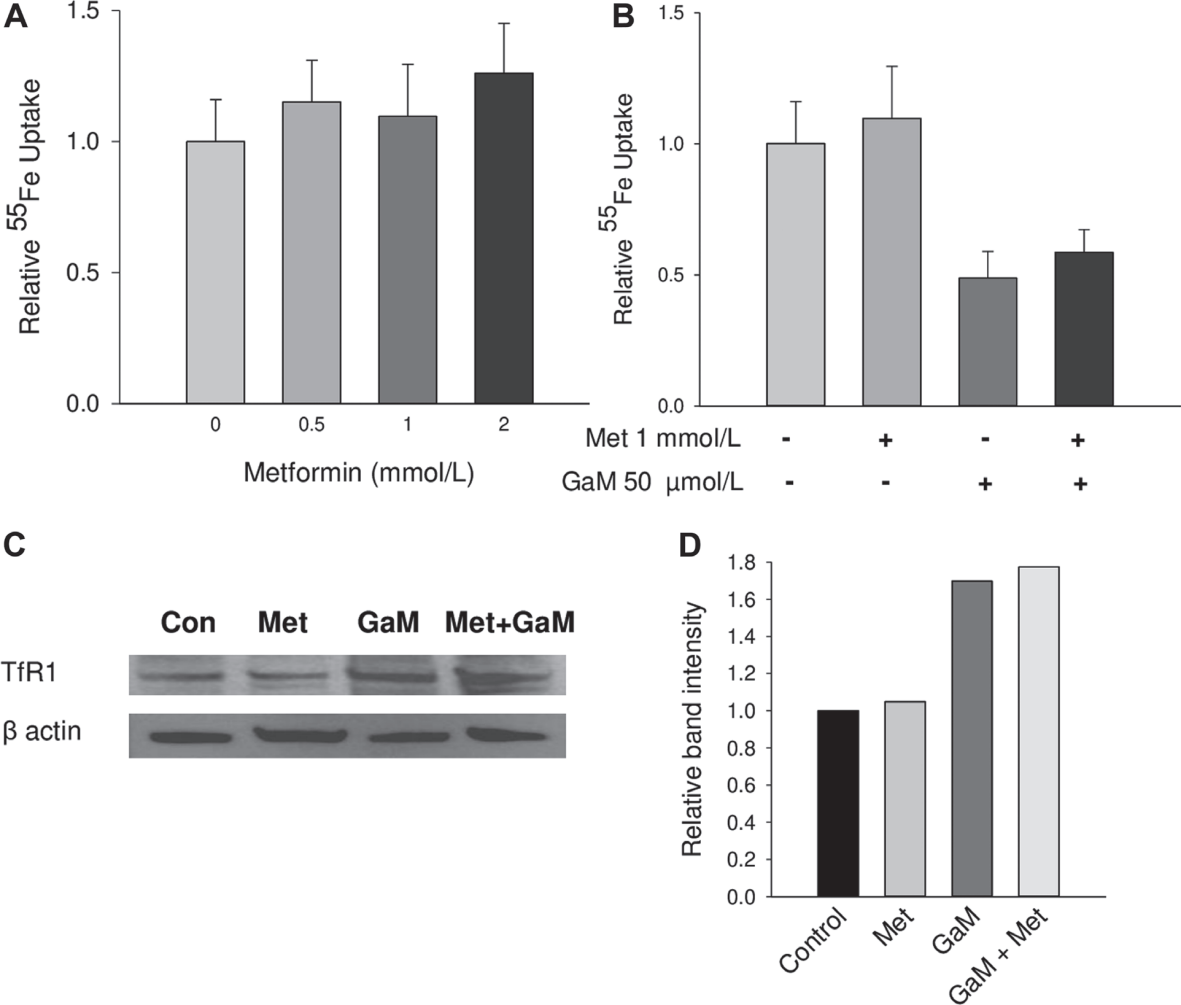
Cellular iron uptake and transferrin receptor1 (TfR1) expression (**A**). Metformin does not affect cellular iron uptake. D54 cells were incubated with increasing concentrations of metformin and ^55^Fe-Tf uptake by cells was measured over a 24 h incubation. (**B**) GaM-inhibition of cellular iron uptake is not affected by metformin. Iron uptake by cells was measured over a 24-h incubation. (**C**) GaM upregulation of TfR1 is not affected by metformin. TfR1 expression was measured by Western blotting. (**D**) Band intensities of TfR1 expression on the Western blot in shown in C.

The mechanisms of gallium’s cytotoxicity include inhibition of cellular iron uptake resulting in relative iron deprivation and a direct interaction of gallium with iron-containing proteins. Collectively, this disrupts cellular iron homeostasis leading to cell death [6]. We therefore examined whether metformin might sensitize cells to GaM’s cytotoxicity by enhancing gallium’s inhibitory action on iron metabolism. However, as shown in Figure 6A, iron uptake by D54 cells was not significantly altered by various concentrations of metformin. Consistent with our earlier studies, GaM inhibited cellular iron uptake but this was not affected by metformin (Figure 6B). GaM’s inhibition of cellular iron uptake and the resultant diminution in cellular iron status result in an increase in TfR1 synthesis [25]. A decrease in cellular iron status increases the interaction of cytoplasmic iron regulatory protein-1 with iron response elements present on the 3′ end of TfR1 mRNA; this results in an upregulation of TfR1 mRNA translation [26]. As expected, GaM-induced iron deprivation produced an upregulation of TfR1 expression in D54 cells; this was not affected by metformin (Figure 6C and 6D). Collectively, these results suggest that metformin enhances GaM’s cytotoxicity in glioblastoma cells through mechanisms that are independent of action on iron metabolism.

### Metformin does not increase the cytotoxicity of an RR inhibitor

**Figure 7:**
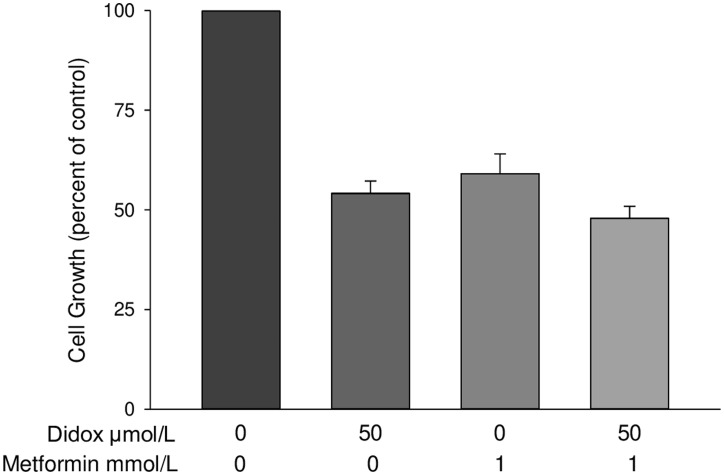
Metformin does not enhance the cytotoxicity of the RR inhibitor Didox. D54 glioblastoma cells were incubated with GaM and metformin alone or in combination at the concentrations shown. Cellular proliferation was measured by MTT assay after a 96-h incubation. Values represent means ± S. E (*n* = 3).

Since our prior studies have shown that a mechanism of gallium’s antineoplastic action includes inhibition of RR [27–29], we questioned whether metformin would enhance the cytotoxicity of Didox, a known inhibitor of RR [30, 31]. However, as shown in Figure 7, while both Didox and metformin alone at the concentrations shown inhibited the proliferation of D54 glioblastoma cells, their combination metformin did not produce a significant further decrease in cell proliferation. Furthermore, in additional experiments using different concentrations of Didox and metformin, we were unable to find synergistic interactions between these drugs (data not shown). These results suggest that metformin enhances GaM’s cytotoxicity in glioblastoma cells through mechanisms that are independent of its action on RR.

## DISCUSSION

The present study is a logical extension of our recent report in which we showed that inhibition of mitochondrial bioenergetics plays an important role in GaM’s antineoplastic activity against glioblastoma *in vitro* and *in vivo* [5]. The intent of the present study was to provide a deeper insight into the mechanisms by which GaM perturbs mitochondrial function. Using XF Analyzer technology, we show for the first time that GaM blocks the activity of mitochondrial complex I in glioblastoma cells and that it does so at a time-point that precedes a decrease in cell proliferation. Our results thus indicate that inhibition of mitochondrial complex I is one of the early events responsible for GaM’s cytotoxicity.

Our results suggest that GaM inhibits complex I activity by interfering with the Fe-S cluster assembly machinery by binding to the scaffold protein IscU [20, 32]. The process of Fe-S cluster assembly and release requires the dynamic IscU scaffold protein to cycle between interchangeable folded and unfolded conformational states [20]. The Fe-S cluster is released from IscU to recipient apoproteins (such as complex I) to generate functional Fe-S cluster-containing holoproteins [19]. We show by NMR spectroscopy that gallium binds IscU and stabilizes its folded state and by a biochemical assay that gallium inhibits Fe-S cluster assembly catalyzed by cysteine desulfurase. Such inhibition could adversely impact normal Fe-S cluster assembly and delivery resulting in a decrease in complex I activity. While inhibition of the Fe-S cluster assembly machinery may be sufficient to block complex I activity, we realize that additional contributory mechanisms could also be at play. For example, others have reported the exchange of Ga for Fe in Fe-S clusters of ferredoxin-thioredoxin reductase and rubedoxin [33, 34]; this could affect the function of those proteins. Beyond a direct action on Fe-S cluster assembly, a GaM-induced block in cellular iron uptake could limit the amount of intracellular iron available for mitochondrial function. Further studies will be needed to dissect the extent to which these latter effects of GaM may contribute to its inhibitory action on complex I.

Our study advances knowledge of the cellular events triggered following exposure of malignant cells to gallium compounds. We previously reported that human lymphoma/leukemia CCRF-CEM cells exhibit a decrease in GSH/GSSG ratio and an increase in ROS production within 1–4 h of incubation with gallium nitrate [21]. That the GaM-induced increase in cellular ROS is of mitochondrial origin was evidenced by the finding that the increase in ROS could be blocked by mitoquinone, a mitochondria-targeted antioxidant [21]. The increase in ROS in Ga-treated cells led to an upregulation of HO-1 expression by triggering the phosphorylation of p38 mitogen-activated protein kinase and activating Nrf-2, a regulator of HO-1 gene expression [21]. While these earlier studies defined a sequence of downstream events arising from gallium-induced cellular ROS production, the upstream events leading to the increase in ROS remained to be determined. Our present study now provides an explanation for the latter and provides a deeper understanding of gallium’s mechanism of action as an antineoplastic agent.

Having shown that GaM can inhibit mitochondrial function through action on complex I, we explored whether its antineoplastic activity could be enhanced by other agents known to inhibit this mitochondrial complex. We chose to examine the effects of metformin on GaM’s cytotoxicity in glioblastoma cell lines because metformin is an FDA-approved oral drug for the treatment of T2DM and its safety and use in the clinic is well established. Metformin blocks complex I activity by binding to its amphipathic region at the interface of its hydrophilic and membrane domains thereby rendering the enzyme in a catalytically inactive conformation which is unable to reduce ubiquinone [14]. Metformin has also shown antineoplastic activity in preclinical studies and a number of clinical trials are underway to evaluate its anti-tumor activity as a single agent or as an adjunct to conventional cancer chemotherapeutic agents [35].

Using both 2D and 3D cell culture systems, we show that a combination of GaM and metformin synergistically inhibit glioblastoma cell proliferation in established glioblastoma cell lines and neurosphere formation in GSCs. It is important to note that marked synergistic cytotoxicity between both agents was seen at concentrations of GaM (25 μmol/L) and metformin (0.5 mmol/L) that in themselves only minimally inhibited cell proliferation (Figure 3D). In addition, the lower concentration of metformin used in this experiment is relevant because other preclinical studies have required much higher greater metformin concentrations to demonstrate its antineoplastic activity [36]. Importantly, our results suggest that the antineoplastic activity of GaM may be optimized when it is combined with another agent that also inhibits mitochondrial function.

Regarding other mechanisms that contribute to drug synergy, we have shown that GaM’s mechanisms of cytotoxicity include interference with cellular iron transport and homeostasis; however, our studies did not reveal an effect of metformin on iron uptake or TfR1 expression. Thus, the synergistic cytotoxicity of GaM and metformin is not due to potentiation (by metformin) of GaM’s action on iron metabolism. Next, we entertained the possibility that metformin might enhance GaM’s inhibitory action on RR; however metformin did not enhance the cytotoxicity of the RR inhibitor Didox. Thus, we conclude that the synergistic cytotoxicity between GaM and metformin is not due to combined action on RR. Collectively, these results support our notion that the combined synergistic inhibition of complex I by GaM and metformin occurs through different sites of action; i. e. interference with Fe-S cluster assembly (for GaM) and binding to complex I (for metformin).

In our investigation, we have further elucidated GaM’s mechanism of action on the mitochondria. As single agents both GaM and metformin have already shown antineoplastic activity in animal tumor models, including glioblastoma [5, 9–11, 37, 38]. Their combination is clinically attractive since metformin is administered orally and can cross the blood brain barrier [39], while oral GaM shows good bioavailabilty in human studies [15].

Emerging data suggest that mitochondrial bioenergetics is a potentially relevant target for cancer therapeutics [40, 41]. Aberrant signaling pathways that trigger tumorigenesis depend on mitochondrial function to support the growth, invasiveness, and metastatic potential of malignant cells [40, 41]. Thus, novel agents that can selectively target tumor mitochondria will add significantly to our therapeutic armamentarium in cancer [42]. Further studies will validate the efficacy of GaM and metformin as a mitochondria-targeting combination *in vivo* and will explore whether other agents that block mitochondrial function can sensitize malignant cells to gallium-based drugs.

## MATERIALS AND METHODS

### Materials

Gallium maltolate was obtained from Titan Pharmaceuticals (South San Francisco, CA). Human transferrin (Tf), 3-(4,5-Dimethylthiazol-2-yl)-2,5-diphenyltetrazolium bromide (MTT), oligomycin, carbonilcyanide 4-(trifluoromethoxy) phenylhydrazone (FCCP), antimycin A, and 1.1-dimethylbiguanide hydrochloride (metformin) were obtained from Sigma Chemical Company (St. Louis, MO). Antibodies to transferrin receptor1 (TfR1), heme oxygenase-1 (HO-1), and beta-actin were purchased from Santa Cruz Biotechnology Inc. (Santa Cruz, CA), while 6-carboxy-2′,7′-dichlorodihydrofluorescein diacetate, di-acetoxymethyl ester (6-H_2_DCF-AM) was purchased from Invitrogen (Carlsbad CA). Didox (3,4-dihydroxybenzohydroxamic acid), a ribonucleotide reductase inhibitor drug [30], was obtained from Howard L. Elford, PhD (Molecules for Health, Richmond, VA) and was prepared as previously described [43]. ^55^FeCl_3_ was purchased from Perkin Elmer (Richmond, CA) and ^55^Fe-Tf was prepared as previously described [44].

### Cell lines

Tissue culture media and supplements were purchased from Life Technologies™ (Grand Island, NY, USA), unless stated otherwise. All cell lines used were validated at their point of origin. Human glioblastoma U-87 MG and D54 cell lines were obtained from American Type Culture Collection (ATCC®, Manassas, VA) and from Dr. D. Bigner, (Duke University Medical Center, Durham, NC), respectively. U-87 MG cells were grown in MEM with Earle’s salts fortified with 10% FBS and supplemented with 1% sodium pyruvate and 0.1% Gentamicin. D54 cells were grown in Improved MEM with Zn Option, fortified with 10% FBS, and supplemented with 0.1% Gentamicin. The glioblastoma stem cell (GSC) model was developed by isolating GSCs from human glioblastoma via sphere culture in serum-free stem cell medium, and was authenticated under an approved IRB protocol, as previously described [45]. The GSC line designated GSC-44 that generates highly invasive orthotopic tumor xenografts was used in this study and was grown as neurospheres in a serum-free stem cell culture medium [24, 45].

### Cytotoxicity

The effects of metformin, GaM, and Didox on cell proliferation *in vitro* were measured by MTT assay as previously described [46]. Cells were plated in culture medium in 96-well plates and incubated for 24 h at 37° C in a CO_2_ incubator. GaM or metformin alone or in combination were added to wells and the incubation continued for an additional 3–7 days. At specified times, 10 μL MTT (5 mg/ml stock solution) was added to each well and cells were incubated at 37° C for an additional 4 h. Cells were solubilized by the addition of 200 μl of 0.04 N HCl in isopropyl alcohol. Similar experiments were run with combination metformin and Didox. The absorbance of each well was determined spectrophotometrically at 570 nm using EL-X808 ultra-microplate auto reader (Biotech Instruments, Winooski, VT) and the absorbance of the wells containing additives was compared with that of the wells without additives (control). Drug interactions were evaluated for synergy as described by Chou and Talalay [23] using a computer program (Dose-effect analysis with microcomputers by J. Chou and T-C Chou, Biosoft, Cambridge, UK).

### Respiratory enzyme activity in intact and permeabilized cells

D54 cells were incubated in 96-well microplates at a density of 7000 cells per well without (control) or with GaM, metformin, or a combination of both agents. After 24 h, at which time cells had attained >95% confluency, the media in the wells was exchanged with fresh media, and mitochondrial function in intact and permeabilized cells was measured using a Seahorse XF96 Extracellular Flux Analyzer (Seahorse Bioscience, North Billerica, MA). Measurement of mitochondrial respiratory complexes in permeabilized cells was performed according to the manufacturer’s instructions, and as detailed by Salabei *et al* [47]. Briefly, intact cells were permeabilized using 1 nmol/L plasma membrane permeabilizer reagent (PMP, Seahorse Bioscience) immediately before oxygen consumption rate (OCR) measurement. The oxygen consumption derived from mitochondrial complex I was measured by providing pyruvate/malate (10 mmol/L and 2 mmol/L, respectively) as substrates for mitochondrial complex 1 while rotenone (1 μmole/L) and antimycin A (10 μmol/L) were used as specific inhibitors of mitochondrial complex I. Results were normalized by measuring the protein content of cells in each well and the oxygen consumption rates were expressed as pmol/min/μg protein.

### Measurement of reactive oxygen species in cells

Intracellular ROS was measured by 2′,7′-dichlorofluorescein (DCF) fluorescence assay, as previously described [48]. D54 and U87 MG cells (5 × 10^5^ cells/ml) were incubated in 96-well white plates for 24 h. 6-carboxy-DCF-AM (10 μmol/L) was then added to the wells to load cells with this compound. After 1 h, GaM 50 μmol/L, metformin 1 mmol/L, or both drugs in combination, were added to the wells and, following an additional 2 or 4 hours of incubation, the medium containing the additives was removed and wells were washed twice with PBS to remove additives and 100 μl of cold PBS was added to each well. DCF fluorescence in each well was measured using an excitation wavelength of 495 nm and emission wavelength of 525 nm in a Perkin Elmer 96-well plate reader. Fluorescent readings were obtained using a FLUOstar Omega Microplate Reader (BMG Labtech, Cary, NC).

### Western blotting

TfR1 and HO-1protein was measured by Western blotting using standard protocols. Cell lysates in sample buffer were resolved by SDS-PAGE and transferred onto a polyvinylidene difluoride membrane using a Blot™ Mini Blot module system (Life Technologies, Carlsbad, CA). Membranes were incubated with primary antibodies against TfR or HO-1 followed by horseradish peroxidase-labeled secondary antibody. Membranes were developed in Enhanced Chemiluminescence Western blotting detection solution (Amersham, Arlington Heights, IL) and exposed to Li-Core scanner.

### Iron-sulfur cluster assembly

We used NMR spectroscopy to investigate the effect of GaCl_3_ on IscU, the scaffold protein on which iron sulfur clusters are assembled. *Escherichia coli* IscU labeled uniformly with nitrogen-15 was prepared as described earlier [49]. We utilized an *in vitro* iron-sulfur cluster assembly reaction to monitor the effect of GaCl_3_ on cluster assembly [50].

### Cellular ^55^FeTf uptake

Iron uptake by D54 cells in the presence of GaM and metformin was measured using ^55^FeTf, as previously described [51].
